# Molecular layer interneurons shape the spike activity of cerebellar Purkinje cells

**DOI:** 10.1038/s41598-018-38264-1

**Published:** 2019-02-11

**Authors:** Amanda M. Brown, Marife Arancillo, Tao Lin, Daniel R. Catt, Joy Zhou, Elizabeth P. Lackey, Trace L. Stay, Zhongyuan Zuo, Joshua J. White, Roy V. Sillitoe

**Affiliations:** 1 Department of Pathology and Immunology, Baylor College of Medicine, 1 Baylor Plaza, Houston, Texas 77030 USA; 2 Department of Neuroscience, Baylor College of Medicine, 1 Baylor Plaza, Houston, Texas 77030 USA; 3 Program in Developmental Biology, Baylor College of Medicine, 1 Baylor Plaza, Houston, Texas 77030 USA; 4 Jan and Dan Duncan Neurological Research Institute of Texas Children’s Hospital, 1250 Moursund Street, Suite 1325, Houston, Texas 77030 USA

## Abstract

Purkinje cells receive synaptic input from several classes of interneurons. Here, we address the roles of inhibitory molecular layer interneurons in establishing Purkinje cell function *in vivo*. Using conditional genetics approaches in mice, we compare how the lack of stellate cell versus basket cell GABAergic neurotransmission sculpts the firing properties of Purkinje cells. We take advantage of an inducible *Ascl1*^*CreER*^ allele to spatially and temporally target the deletion of the vesicular GABA transporter, *Vgat*, in developing neurons. Selective depletion of basket cell GABAergic neurotransmission increases the frequency of Purkinje cell simple spike firing and decreases the frequency of complex spike firing in adult behaving mice. In contrast, lack of stellate cell communication increases the regularity of Purkinje cell simple spike firing while increasing the frequency of complex spike firing. Our data uncover complementary roles for molecular layer interneurons in shaping the rate and pattern of Purkinje cell activity *in vivo*.

## Introduction

**Figure 1:**
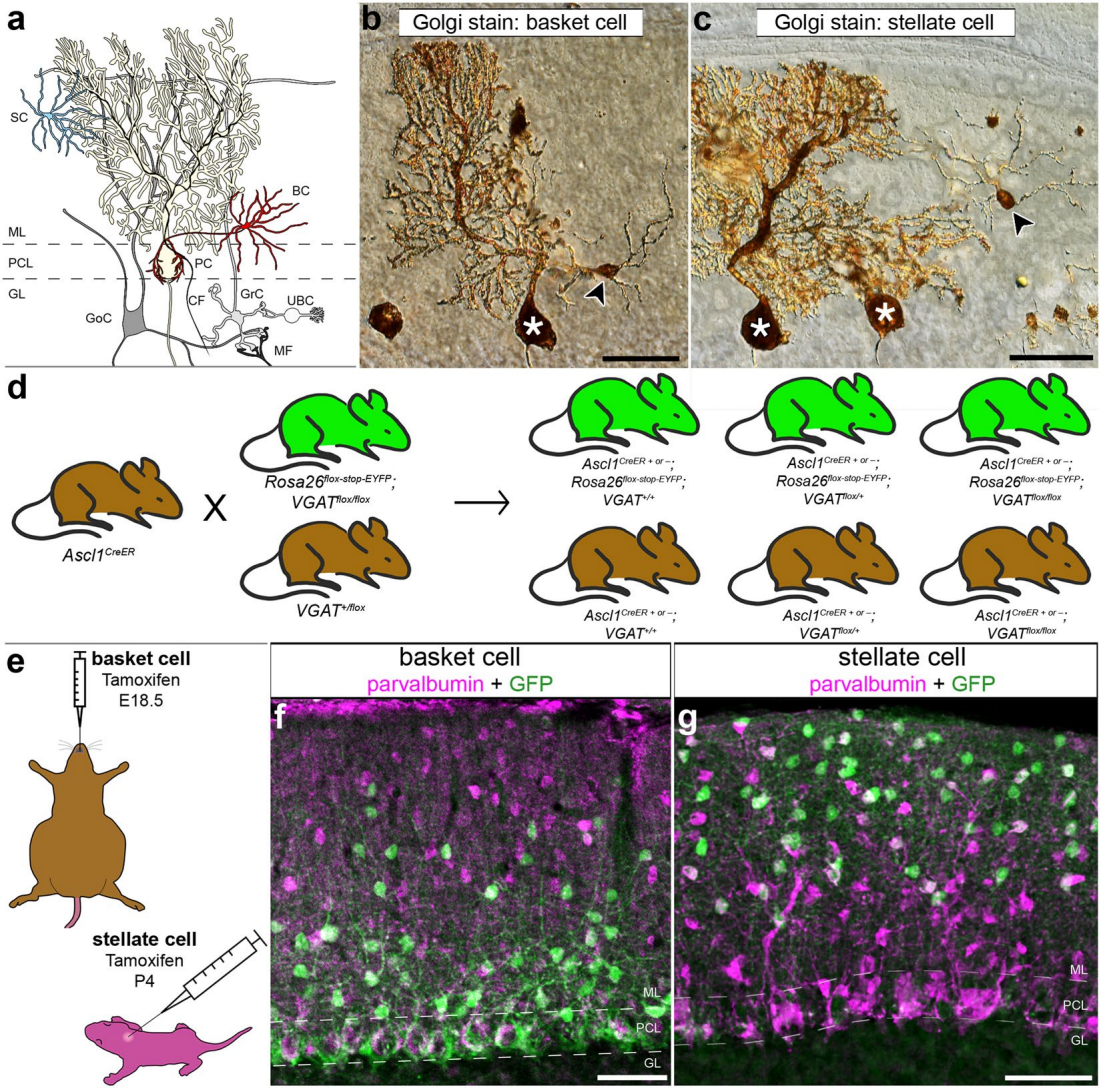
The *Ascl1*^*CreER*^ allele can be used for genetic marking of stellate cells and basket cells. (**a**) Schematic of cerebellar circuitry. The Purkinje cell (PC), basket cell (BC), and stellate cell (SC) are colorized while other cells and fibers in the cerebellar cortex are represented in grayscale (climbing fiber = CF, mossy fiber = MF, Golgi cell = GoC, granule cell = GrC, unipolar brush cell = UBC). Dotted lines represent the borders of the Purkinje cell layer (PCL) with the molecular layer (ML) and granule cell layer (GL). (**b,c**) Golgi-Cox stain of cerebellar tissue. Scale = 50 μm. (**b**) Basket cell (arrowhead) and Purkinje cell (asterisk) revealed by Golgi-Cox stain. (**c**) Stellate cell (arrowhead) and Purkinje cells (asterisks) revealed by Golgi-Cox stain. (**d**) Representation of breeding scheme. (**e**) Schematic of methods for tamoxifen administration. Tamoxifen was administered via oral gavage to pregnant dams at E18.5 to achieve constitutive marking and manipulation of a subset of basket cells in the resulting pups (upper left). Tamoxifen was administered via subcutaneous injection into the scruff of pups at P4 to achieve constitutive marking and manipulation of a subset of stellate cells (bottom right). (**f**) Labeled cells were found in the basal molecular layer in animals treated with tamoxifen at the basket cell timepoint and the apical molecular layer for those treated at the stellate cell timepoint (**g**). Scale = 50 μm. 5 sections separated by ~200 µm around midline per mouse, N = **7** for each condition.

The cerebellum is essential for diverse motor functions including coordination, learning, posture, and balance^1^. Despite this functional diversity, a core cerebellar circuit mediates all of its functions^2,3^. This canonical cerebellar circuit is comprised of relatively few types of cells^4^. The Purkinje cells, the sole output of the cerebellar cortex and main computational cell type, are located at the center of the circuit (Fig. 1a). Purkinje cells receive input from several classes of interneurons. The granule cells project parallel fibers that send excitatory signals to Purkinje cells^5–8^. However, in the posterior cerebellum, the unipolar brush cell interneurons can influence granule cell output by amplifying vestibular inputs that are delivered to the cerebellum by mossy fibers^9^. Golgi cells, another cell type of the granular layer, interact with granule cells and mediate feedforward and feedback signaling in the cerebellar cortex^10,11^. Purkinje cells also receive direct inhibitory inputs from basket cells that form pericellular baskets as well as specialized terminals known as pinceaux, and also from stellate cells that terminate on the smooth shafts of the Purkinje cell dendrites (Fig. 1b,c)^12^. Together, the different classes of interneurons play an essential role in controlling cerebellar cortical output during motor behavior^13^. However, how each class of interneurons influences Purkinje cell firing is poorly understood. Here, we used inducible conditional genetic approaches in mice to test whether the two classes of cerebellar molecular layer interneurons have dedicated GABAergic functions *in vivo*.

Cerebellar interneurons come from distinct lineages and have specific birth dates^14–17^. Fate mapping and transplant experiments demonstrated that the inhibitory interneurons are generated in a precise spatial and temporal manner such that the early born neurons occupy deep positions within the cerebellar cortex whereas later born neurons migrate to the more superficial locations^18–20^. More recent genetic inducible fate mapping experiments corroborated those results, and further suggested that the timing of *Ascl1* gene expression during differentiation may be used as a molecular time stamp for the birth of specific classes of GABAergic interneurons^21^. *Ascl1*, also known as *Mash1*, is a basic helix-loop-helix transcription factor that is expressed during cerebellar development^21,22^. In this study, we used the *Ascl1*^*CreER*^ genetic fate-mapping allele^21^ to not only mark interneurons, but also to constitutively silence their output. To do so, we selectively deleted a critical functional domain in the *Vgat* gene^23^, which removed the ability of the inhibitory interneurons to signal their output using fast GABAergic neurotransmission. Genetic deletion using *Ascl1*^*CreER*^ allowed us to independently target newly differentiated stellate cell and basket cell interneurons in the molecular layer because these neurons are born at different stages of cerebellar development, and intriguingly almost exclusively during the peri- to post-natal period when the cerebellar circuits are wiring up for function^24^. This is advantageous for our study because *in vitro* studies showed that as development progresses, interneuron to Purkinje cell inhibition increases^25^. Functional studies support these data since removing the interneurons or their postsynaptic γ2 GABA(A) receptors obstruct motor learning^26,27^. Recent work also demonstrates that movement rate is dependent on coordinated molecular layer interneuron activity^28^. Still, there is a long-standing debate as to whether stellate cells and basket cells are distinct types of interneurons^29,30^, and more broadly whether they perform different functions in the cerebellar circuit^31^. In this study, we genetically mark stellate cells and basket cells independently and manipulate their GABAergic neurotransmission as the cells are born to determine their impact on establishing the mature firing properties of Purkinje cells *in vivo*.

## Results

### A mouse genetic strategy for marking and manipulating cerebellar GABA interneurons

**Figure 2:**
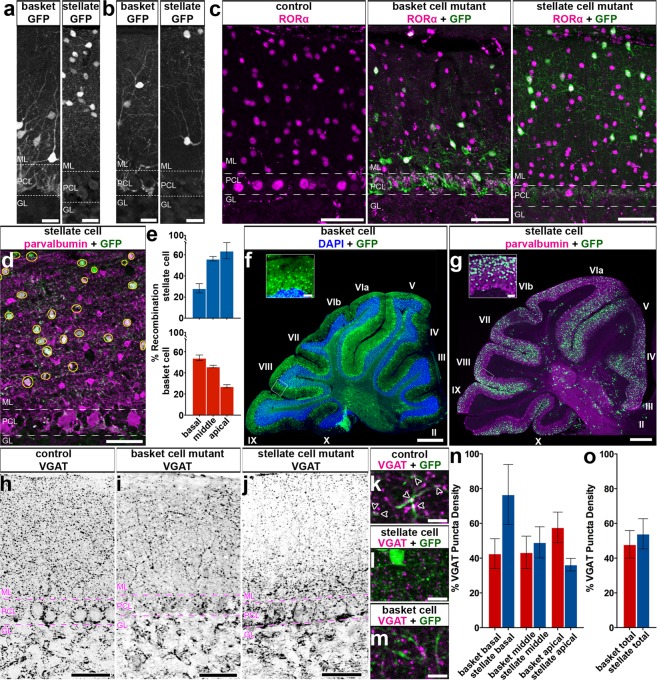
*Ascl1*^*CreER*^ conditional deletion of VGAT protein is efficient and selective. (**a,b**) Labeled cells in the basket and stellate conditions. Scale = 20 μm. 5 sections separated by ~200 µm around midline were analyzed per mouse, N = **7** for each condition. (**a**) Labeled basket cells (left) and labeled stellate cells (right) in customary regions of the ML. (**b**) Labeled basket cells (left) and stellate cells (right) farther from their traditional regions of the ML. (**c**) RORα expression in the ML of control (left), basket cell mutant (middle), and stellate cell mutant (right) mice. Scale = 50 μm. Per condition: N = 3, n = 9. (**d**) Representation of recombination quantification. GFP-expressing cells were counted (yellow circles) and compared to a count of the total number of ML interneurons. Scale = 50 μm. (**e**) Quantification of recombination efficiency in basket and stellate cell conditions. (**f,g**) Sample of a whole sagittal cerebellar section in the basket (**f**) and stellate (**g**) cell manipulation conditions. White boxes indicate the regions that are shown as blow-ups in the insets. Cerebellar lobules are indicated with Roman numerals. Scale = 0.5 mm. Inset scale = 50 μm. (**h–j**) VGAT expression across the ML in control mice (**h**), basket cell VGAT deletion mice (**i**) and stellate cell VGAT deletion mice (**j**). Scale = 50 μm. (**k–m**) Putative synapses where VGAT and GFP overlapped (arrowheads) could be readily found in control tissue (**k**), but were absent or not readily found in the apical molecular layer of mutant stellate cell tissue (**l**) as well as the basal molecular layer of mutant basket cell tissue (**m**). Scale = 10 μm. Control N = 3, n ≥ 3; basket cell mutant: N = 2, n ≥ 3; stellate cell mutant: N = 6, n ≥ 3. (**n**) Quantification of VGAT puncta density in the basal, middle, and apical ML of basket and stellate VGAT mutant mice. (**o**) Quantification of VGAT puncta density in the entire ML in both mutant conditions. (**a–d,h–j**) Dotted lines indicate the borders of the Purkinje cell layer (PCL) with the molecular layer (ML) above and the granular layer (GL) below.

We aimed to manipulate neurotransmission in a way that would block the activity of the molecular layer interneurons without inducing changes in cerebellar morphology or causing neurodegeneration. We therefore targeted the function of the vesicular GABA transporter (VGAT), a transporter that is essential for the uptake of GABA into synaptic vesicles. Conditional knockout of *Vgat* in Purkinje cells does not induce widespread defects in cerebellar anatomy^32^, making it an ideal target for genetic deletion. We targeted the removal of the *Vgat* gene in stellate cells and basket cells in the cerebellar cortex by using the *Mash1/Ascl1* promoter to drive tamoxifen-inducible Cre in the cerebellum (Fig. 1d)^21^. The *Mash1/Ascl1* gene (referred to from here on as *Ascl1*) encodes a developmental transcription factor that is critical for the specification of neurons and glia^22^. In the cerebellum, it is expressed in waves by neural and glial precursors as cells exit the cell cycle and begin to differentiate^21^. The period of stellate cell differentiation begins at late embryonic stages and reaches peak levels at postnatal day (P) 3 - P5 whereas basket cell differentiation occurs during late embryogenesis and peaks at around embryonic day (E) 18^21^. Before manipulating *Vgat*, we first tested the genetic strategy by marking cells. To specifically target stellate cells we subcutaneously injected *Ascl1*^*CreER*^*;R26*^*fx-stop-EYFP*^ postnatal pups with a single 20 mg/ml dose of tamoxifen at P4 (Fig. 1e,g), which would allow for recombination in *Ascl1* expressing cells for the next ~32 hours^33^. But note that we predicted to label only subsets of interneurons since they are born over several days. Analysis of the GFP expression showed labeling of neurons in the upper two thirds of the molecular layer (Fig. 1g, 5 sections separated by ~200 µm around midline per mouse, N = **7**). Morphological analysis of individual neurons that were marked by GFP confirmed their “stellate” appearance as well as their pattern of axonal projections within the molecular layer (Figs 1g and 2a). We next confirmed whether we could target putative basket cells, as demonstrated previously using a different reporter^21^. We targeted the reporter to neurons located in the basal one third of the molecular layer by delivering tamoxifen to E18.5 embryos by oral gavage of *Ascl1*^*CreER*^*;R26*^*fx-stop-EYFP*^ pregnant dams (Fig. 1e,f). The morphology of these neurons was consistent with their identity as basket cells, namely because of the presence of baskets on the Purkinje cell somata (Figs 1f, 2a, 5 sections separated by ~200 µm around midline per mouse, N = 7). We could also track their prominent axons that travel in a transverse trajectory within the molecular layer, in close proximity to their targets, the Purkinje cell somata, which are located immediately below the axons (Figs 1f and 2a).

In addition to labeling what would be considered typical stellate cells and basket cells (Fig. 2a), we could also reveal neurons with structural variations, but likely belonging to these same classes. In the stellate cell marking scheme, cells with a more restricted dendritic span were observed in the very apical regions of the molecular layer (Fig. 2a), and within the middle of the layer we could label cells with soma positions that mimicked basket cells (Fig. 2b). Regardless of soma position, when the stellate cell marking scheme is used to remove *Vgat*, the predominant loss of VGAT expression in the deletion allele was always in the more apical locations of the molecular layer (Fig. 2h,j). The basket cell marking scheme also labeled cells in the middle portion of the molecular layer, and these cells projected either ascending or descending processes (Fig. 2a,b). Therefore, although the stellate cells and basket cells, defined strictly by position and density, could be separated using the *Ascl1*^*CreER*^ lineage tracing, each class also contains cells with a varying range of specializations that are observed in their dendritic processes and axonal projections. Thus, neuronal position within the molecular layer alone is not necessarily indicative of the identity of that interneuron, or the specific interneuron cell class that it belongs to. However, the cellular anatomy revealed by our genetic marking data are consistent with the results of classic Golgi staining of molecular layer interneurons^34^.

### The *Ascl1*^*CreER*^ allele has high specificity and recombination efficiency in interneurons

We next tested whether we could confirm if the labeling of apical and basal molecular layer neurons reflect specifically stellate cells and basket cells, respectively. The reporter expressing cells colocalized with the expression of parvalbumin, which is a well-known marker for Purkinje cells and molecular layer interneurons (Figs 1f,g and 2d)^35^. We did not detect any GFP labeling in parvalbumin-immunoreactive Purkinje cells (Figs 1f,g and 2d), which is consistent with the earlier marking of Purkinje cells with *Ascl1*^*CreER*^ between E10 and E13^21^. The distribution of reporter expression in stellate versus basket cells was validated by RAR-related orphan receptor alpha (RORα) expression (Fig. 2c, per condition: N = 3, n = 9), which also marks molecular layer interneurons and Purkinje cells^14,36–38^. An advantage of using RORα expression, a nuclear hormone receptor, is that the cytoplasmic GFP labeling in marked neurons pairs nicely with the robust staining of the nucleus. The adult stellate cells that were marked by giving pups tamoxifen at P4 expressed RORα, as did the adult basket cells that were marked at E18.5 (Fig. 2c middle and right). Similar to parvalbumin, when we used RORα expression as a marker we did not detect GFP in Purkinje cells (Fig. 2c middle and right). Moreover, we did not detect GFP expression in any of the granular layer interneurons (Fig. 2c middle and right). We conclude that our *Ascl1*^*CreER*^ genetic marking schemes are selective for the classes of inhibitory interneurons that reside within the molecular layer. With consideration of these classes’ date of differentiation, morphology, layer location, and protein expression profile, for the remaining duration of this text, the cells that are marked using the E18.5 and P4 induction time points will be referred to as “basket cells” and “stellate cells,” respectively.

The efficiency of *Ascl1*^*CreER*^ recombination on the *R26*^*fx-stop-EYFP*^ reporter allele provides a prediction for the percentage of interneurons that can be manipulated with this genetic paradigm. It was essential to estimate how widespread and reliable the cell marking strategy is before crossing the *Ascl1*^*CreER*^ line to a functional allele such as *Vgat*^*fx/fx*^ for testing circuit function. We quantitatively examined the number of parvalbumin or RORα expressing molecular layer interneurons that also express GFP reporter in *Ascl1*^*CreER*^*;R26*^*fx-stop-EYFP*^ mice (Fig. 2d). We found that, while many of these cells coexpressed GFP, some GFP-expressing cells with neuronal morphology did not coexpress either parvalbumin or RORα. This is not surprising as there is no perfect marker for molecular layer interneurons^30^. However, we viewed coexpression with these markers as an underestimate of recombination efficiency. Using this expected underestimate, we found that recombination was highest in the basal molecular layer in the basket cell marking scheme and highest in the apical molecular layer in the stellate cell marking scheme (stellate: basal = 19.73% ± 4.72%, middle = 42.47% ± 1.12%, apical = 46.05% ± 11.26%; basket: basal = 46.84% ± 2.13%, middle = 40.40% ± 3.25%, apical = 22.37% ± 0.72%). To establish an upper bound on the recombination efficiency, we included the number of GFP-only expressing cells in our calculation. We found that in the stellate cell marking scheme the majority of labeled cells were in the apical molecular layer (basal = 28.35% ± 4.41%, middle = 56.30% ± 2.14%, apical = 63.90% ± 7.70%; N = 3, n = 6) while in the basket cell marking scheme the majority of the labeled cells were in the basal molecular layer (basal = 54.19% ± 2.70%, middle = 46.00% ± 1.23%, apical = 27.27% ± 1.51%; N = 3, n = 9) (Fig. 2e). Likely, the recombination efficiency of our method is between these bounds. This percent recombination marked enough putative basket cells to project axons that form baskets on almost every Purkinje cell in the field of view, on any given tissue section (Figs 1f, 2c middle). Of importance to visualizing the lineage of interneuron classes, we were successful in marking a similar number of cells, overall, in both conditions while avoiding unwanted recombination throughout the entire molecular layer. These features allowed class specific interneuron targeting plus accommodated the need to make comparisons between the stellate cells and basket cells (total molecular layer coexpression: stellate scheme lower bound = 36.95% ± 4.31%, basket scheme lower bound = 36.88% ± 1.32%, *P* = 0.99; stellate scheme upper bound = 52.17% ± 4.06%, basket scheme upper bound = 43.28% ± 1.43%, *P* = 0.15). This marking is also sufficient for distinguishing the relative distributions of neurons that contribute to the molecular layer populations. Additionally, in both genetic marking paradigms, we could detect GFP reporter expression in all lobules of the cerebellum, and we were able to mark neurons in the vermis, paravermis, and also in the hemispheres (Fig. 2f,g, N = 7 per condition). Therefore, we did not find systematic regional biases in the localization of interneuron populations that were targeted by our genetic marking paradigms.

### Targeted loss of VGAT protein in conditional *Ascl1*^*CreER/*+^*; Vgat*^*fx/fx*^ mutant mice

We set up crosses to generate litters with genotype *Ascl1*^*CreER/*+^*;R26*^*fx-stop-EYFP*^*;Vgat*^+*/*+^ (control) and genotype *Ascl1*^*CreER/*+^*;R26*^*fx-stop-EYFP*^*;Vgat*^*fx/fx*^ (mutant). This approach allows us to mark and manipulate the same neurons *in vivo*. After tamoxifen treatment at P4, we expected to mark and manipulate stellate cells in the mutants and only mark cells in the control. We expected a similar manipulation for basket cells after providing tamoxifen at E18.5. To test whether VGAT was removed from the intended neurons we quantified the number and distribution of VGAT-positive synaptic terminals in the molecular layer. VGAT expression in the molecular layer of control mice showed an approximately uniform distribution of punctae from the basal to the apical regions (Fig. 2h). Stellate cells, basket cells, and Purkinje cell axon collaterals are the main contributors to the GABAergic synapses marked by VGAT expression in the molecular layer. We found that the density of VGAT punctae in the stellate cell mutant mice was significantly reduced, specifically in the apical region of the molecular layer (Fig. 2j,n; mean VGAT density as percent of control: apical = 36.26% ± 3.62, *P* = 0.03; middle = 49.08% ± 9.00, *P = *0.07; basal = 76.63% ± 17.23, *P* = 0.43; stellate mutant: N = 3, n = 3; stellate control: N = 2, n = 3). In contrast, after deleting *Vgat* in basket cells, we found significantly reduced expression of VGAT in the basal portion of the molecular layer, but we also found a marked reduction, albeit less pronounced, in the middle and apical regions (Fig. 2i,n; mean VGAT density as percent of control: apical = 57.69% ± 8.80, *P* = 0.10; middle = 43.33% ± 9.32, *P* = 0.05; basal = 42.62% ± 8.56, *P* = 0.03, per condition: N = 3, n = 9). Loss of basal VGAT expression is due to manipulation of the baskets and pinceaux whereas loss of VGAT apically is due to manipulation of basket cell synapses made by the ascending collateral axons (Fig. 2i, see 2a,b and 1b)^34^. Importantly, expression of VGAT and GFP overlapped at putative inhibitory synapses in control animals (Fig. 2k), but a loss of overlap in VGAT and GFP expression was noted in the apical molecular layer in the stellate cell mutant condition and in the basal molecular layer in the basket cell mutant condition, reflecting the selective removal of VGAT from these cells (Fig. 2l,m). Interestingly, total VGAT expression in the molecular layer was not significantly different between the basket cell and stellate cell manipulations (Fig. 2o; basket cell mean VGAT density as percent of control = 47.95 ± 7.96, per condition: N = 3, n = 9; stellate mean VGAT density as percent of control = 54.03 ± 8.70, stellate mutant: N = 3, n = 3; stellate control: N = 2, n = 2; *P* = 0.63). These data confirm that genetic deletion of *Vgat* with *Ascl1*^*CreER*^ is effective for manipulating VGAT protein. The data also show that the *Ascl1*^*CreER*^ allele can be used for region-specific deletion of VGAT in a cerebellar layer where classes of related neurons are co-residing.

### Deletion of *Vgat* does not prevent interneurons from occupying the molecular layer

Deletion of *Vgat* could result in a loss of VGAT because the protein is depleted or because cells are lost. Indeed deletion of genes encoding for molecules involved in neurotransmission can result in cerebellar cell death^39^, and notably when these molecules are expressed during development^40–42^. To test this possibility, we again stained for the nuclear hormone receptor, RORα, to visualize interneuron distribution in lobule III or IV. Lobules III and IV are ideal for systematically examining molecular layer anatomy because the deep fissures provide long, straight regions of cortex that allow consistent measures for analysis. We found that the density of molecular layer interneurons that express RORα in both the stellate cell and basket cell mutants (Fig. 2c; stellate cells – control = 1.22 × 10^−4^ cells/μm^3^ ± 3.60 × 10^−5^, N = 3, n = 3; mutant = 1.17 × 10^−4^ cells/μm^3^ ± 1.71 × 10^−5^; *P = *0.91, N = 3, n = 9; basket cells – control = 1.14 × 10^−4^ cells/μm^3^ ± 1.14 × 10^−5^, N = 3, n = 3; mutant = 9.56 × 10^−5^ cells/μm^3^ ± 2.21 × 10^−5^, N = 3, n = 9; *P = *0.51) was not significantly different from controls. Therefore, loss of VGAT does not kill the interneurons.

### Loss of *Vgat* in newly differentiated interneurons causes Purkinje cell firing defects

**Figure 3:**
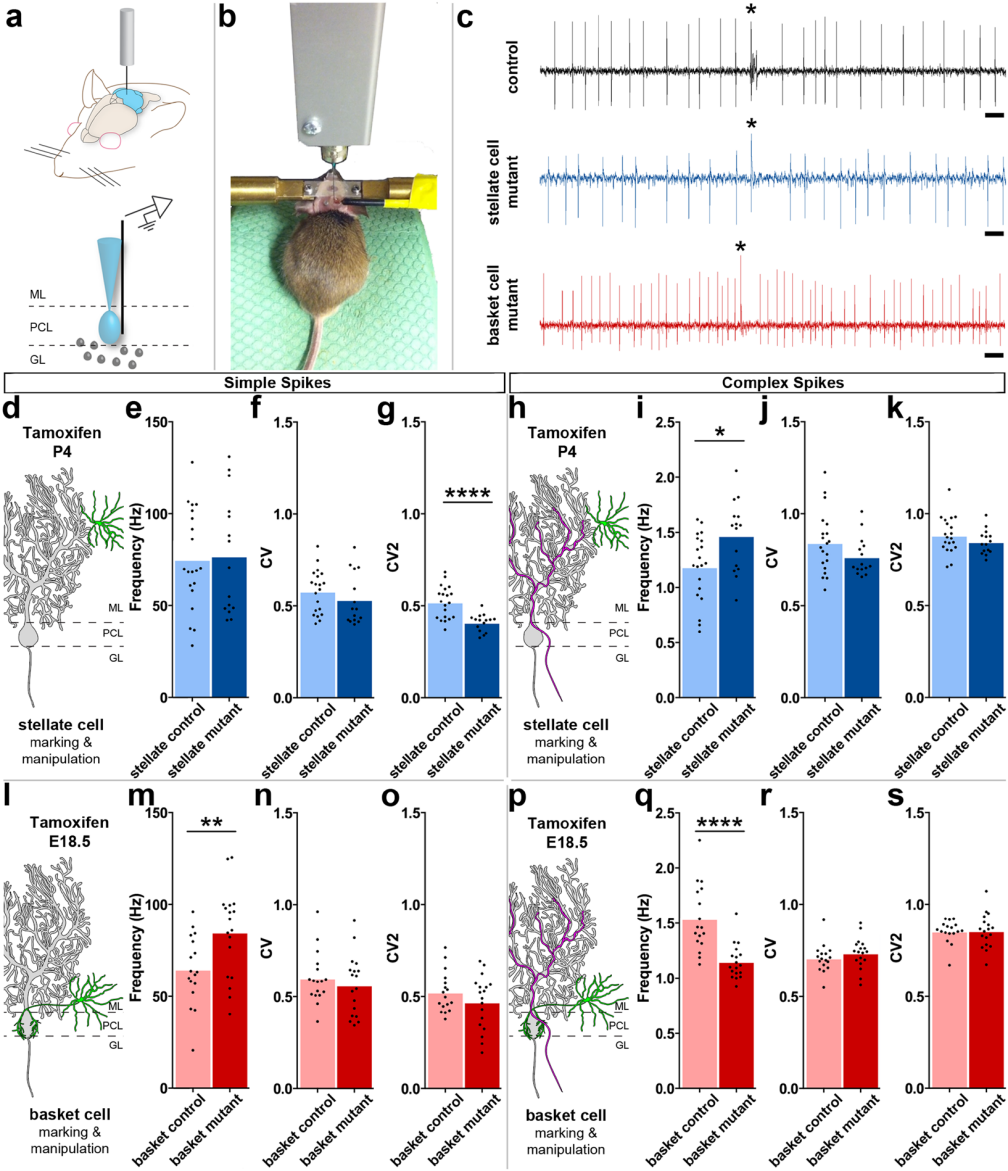
Genetic depletion of GABAergic molecular layer interneuron neurotransmission alters Purkinje cell firing *in vivo*. (**a**) Schematic of electrophysiology setup. ML (molecular layer), PCL (Purkinje cell layer), GL (granular layer) (**b**) Picture of a mouse in the electrophysiology setup. (**c**) Example recordings of Purkinje cells in a control (top), stellate cell mutant (middle), and basket cell mutant (bottom) mouse. Complex spikes indicated with asterisks. Scale = 20 ms. (**d**) Schematic of a stellate cell (green) in relation to a Purkinje cell (grey). (**e–g**) Purkinje simple spike electrophysiology in the stellate cell condition (control: N = 7, n = 20; mutant: N = 3, n = 15). (**e,f**) Firing frequency (**e**) and CV (**f**) were not significantly different. (**g**) CV2 was significantly decreased compared to control. (**h**) Schematic of a climbing fiber (magenta) to a stellate cell (green) and a Purkinje cell (grey). (**i–k**) Purkinje complex spike electrophysiology in the stellate cell condition (control: N = 7, n = 20; mutant: N = 3, n = 15). (**i**) Firing frequency was significantly increased compared to control. (**j,k**) Neither CV (**j**) nor CV2 (**k**) were significantly different. (**l**) Schematic of a basket cell (green) in relation to a Purkinje cell (grey). (**m–o**) Purkinje simple spike electrophysiology in the basket cell condition (control: N = 5, n = 17; mutant: N = 3, n = 18). (**m**) Firing frequency was significantly increased. (**n,o**) Neither CV (**n**) nor CV2 (**o**) were significantly changed. (**p**) Schematic of a climbing fiber (magenta) in relation to a basket cell (green) and a Purkinje cell (grey). (**q–s**) Purkinje cell complex spike electrophysiology in the basket cell condition (control: N = 5, n = 17; mutant: N = 3, n = 18). (**q**) Firing frequency was significantly decreased in mutants compared to control mice. (**r,s**) Neither CV (**r**) nor CV2 (**s**) were significantly changed.

To test for electrophysiology defects we analyzed *Ascl1*^*CreER/*+^*;R26*^*fx-stop-EYFP*^*;Vgat*^+*/*+^ (control) and *Ascl1*^*CreER/*+^*;R26*^*fx-stop-EYFP*^*;Vgat*^*fx/fx*^ mice (mutant). However, *Ascl1*^*CreER/*+^*;Vgat*^*fx/fx*^ mutants without the marking allele were also used for analysis. We performed extracellular single-unit recordings with tungsten electrodes. To access the cerebellum, a craniotomy and recording port were positioned over lobule VI of the vermis (Fig. 3a)^43^. Alert adult mice were allowed to stand on a wheel during recordings (Fig. 3b). Although the mice are free to walk on the wheel, the periods of most stable recordings that were used to quantify the Purkinje cell responses were acquired when the mice were sitting at rest. Therefore, these recordings can be considered to have occurred during quiet wakefulness. Purkinje cells were recorded at a depth of 0–2 mm from the surface of the cerebellum and were identified by their characteristic complex spikes (Fig. 3c). To examine the firing properties of Purkinje cells, we measured both the firing frequency and the variability of the firing pattern in alert mice for both simple spike and complex spike activity. Firing frequency was measured as the mean number of spikes over time, and indicates the level of activity of a cell. The variability of the firing pattern was measured using two parameters: the coefficient of variance (CV), which measures the variability in firing intervals over the entire recording session, and CV2, which measures the variability of firing intervals between two adjacent spikes^44^. Loss of stellate cell GABAergic neurotransmission increases the regularity of Purkinje cell simple spike firing as measured by CV2 (Fig. 3g; control = 0.52 ± 0.02; N = 7, n = 20; mutant = 0.41 ± 0.01; N = 3, n = 15; *P* < 0.0001). However, we did not detect a significant change in CV (Fig. 3f; control = 0.58 ± 0.03; N = 7, n = 20; mutant = 0.53 ± 0.04; N = 3, n = 15; *P* = 0.32) or the firing rate (Fig. 3e; control = 74.99 Hz ± 6.03 Hz; N = 7, n = 20; mutant = 76.85 Hz ± 8.30 Hz; N = 3, n = 15; *P* = 0.86). Interestingly, loss of basket cell GABAergic neurotransmission resulted in an increase in the frequency of Purkinje cell simple spike firing (Fig. 3m; control = 64.56 Hz ± 4.62 Hz; N = 5, n = 17; mutant = 84.76 Hz ± 5.67 Hz; N = 3, n = 18; *P* = 0.01). There was no significant change in CV (Fig. 3n; control = 0.60 ± 0.03; N = 5, n = 17; mutant = 0.56 ± 0.04; N = 3, n = 18; *P* = 0.46) or CV2 (Fig. 3o; control = 0.52 ± 0.03; N = 5, n = 17; mutant = 0.47 ± 0.03; *P* = 0.23). Further, there was a divergent effect of the lack of stellate cell and basket cell GABAergic neurotransmission on complex spike activity. Lack of stellate cell neurotransmission increases the complex spike firing rate (Fig. 3i; control = 1.19 Hz ± 0.07 Hz; N = 7, n = 20; mutant = 1.47 Hz ± 0.08 Hz; N = 3, n = 15; *P = *0.01). This occurs without a significant change in CV (Fig. 3j; control = 0.84 ± 0.04; N = 7, n = 20; mutant = 0.77 ± 0.03; N = 3, n = 15; *P* = 0.11) or CV2 (Fig. 3k; control = 0.88 ± 0.02; N = 7, n = 20; mutant = 0.85 ± 0.02; N = 3, n = 15; *P* = 0.20). However, the lack of basket cell neurotransmission decreases the complex spike firing rate (Fig. 3q; control = 1.54 Hz ± 0.07 Hz; N = 5, n = 17; mutant = 1.15 Hz ± 0.04 Hz; N = 3, n = 18; *P* < 0.0001). This also occurs without a significant change in CV (Fig. 3r; control = 0.71 ± 0.02; N = 5, n = 17; mutant = 0.73 ± 0.02; N = 3, n = 18; *P* = 0.33) or CV2 (Fig. 3s; control = 0.85 ± 0.02; N = 5, n = 17; mutant = 0.86 ± 0.02; N = 3, n = 18; *P* = 0.94). These data suggest that stellate cell and basket cell GABAergic output activity cooperate to establish the proper rate and pattern of simple spike and complex spike firing of Purkinje cells *in vivo*.

### Loss of molecular layer interneuron inhibition does not cause neurodegeneration

**Figure 4:**
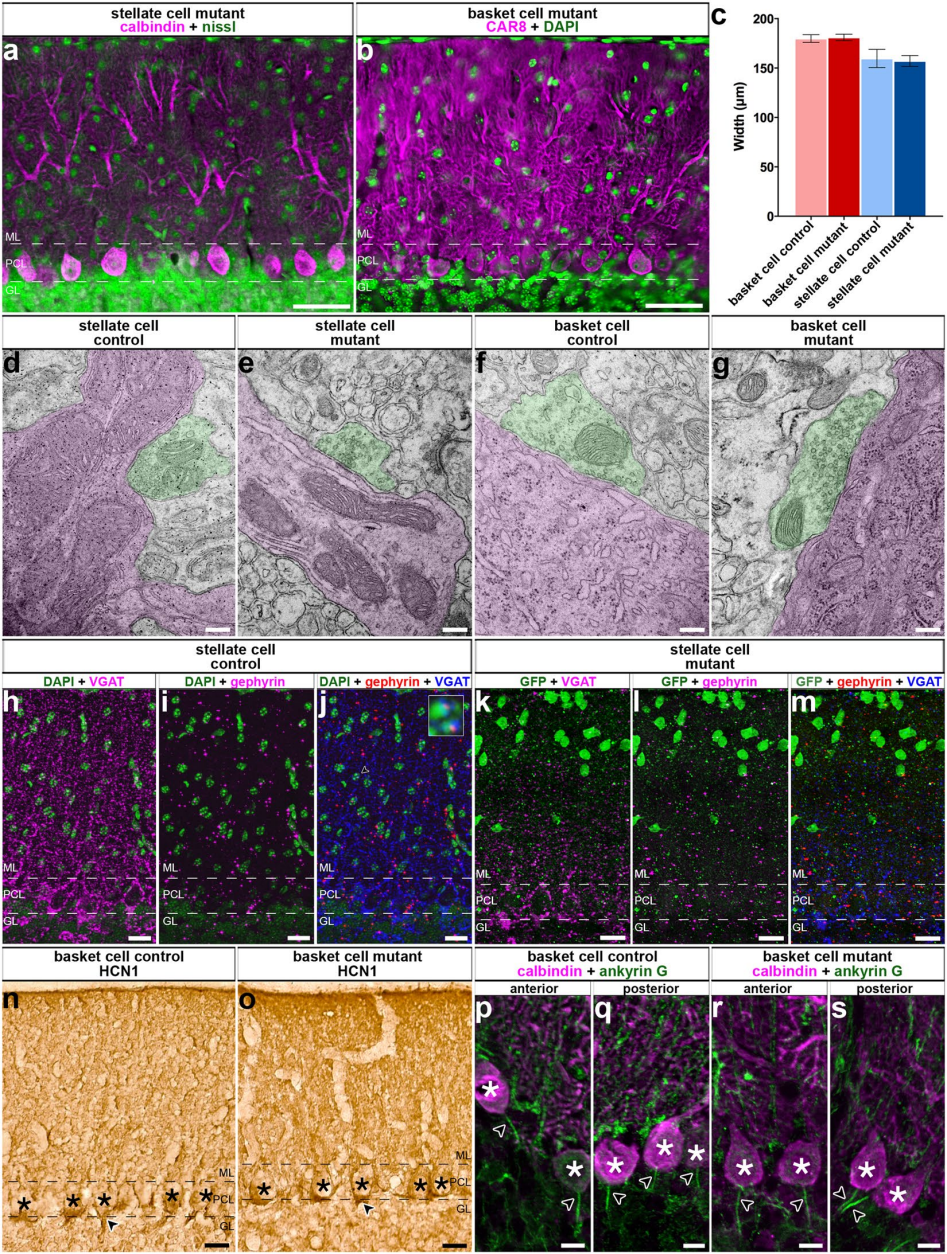
Deleting *Vgat* in molecular layer interneurons does not rearrange cerebellar circuitry or induce neurodegeneration. (**a,b**) Examples of images used for quantification of ML thickness. Purkinje cell layer (PCL), molecular layer (ML), and granular layer (GL). Scale = 50 μm. (**c**) Quantification of ML thickness in all conditions (basket cell control mean = 179.9 μm ± 3.83, N = 3, n = 9, basket cell mutant mean = 181.1 μm ± 3.16, N = 3, n = 9, *P* = 0.82; stellate cell control mean = 159.7 μm ± 9.20, N = 3, n = 18, stellate cell mutant mean = 157.2 μm ± 5.50, N = 6, n = 36, *P* = 0.83). (**d,e**) TEM images revealed normal synapses in all conditions. Purkinje cells and processes are colorized in magenta and putative basket and stellate synaptic terminals are colorized in green. Scale = 200 nm. (**d,e**) Stellate cell: N = 2, n ≥ 10, per condition. (**f,g**) Basket cell: N = 3, n ≥ 9, per condition. (**h–m**) Gephyrin expression was unchanged in stellate cell mutant mice compared to control. Scale = 20 μm. (**h–j**) Control mice (N = 3, n ≥ 3) have uniform expression of VGAT (**h,j**) and gephyrin (**i,j**) in the ML. (**j**) Example triple labeled synapses (arrowhead) are shown in the blowup. (**k–m**) Stellate cell mutant mice do not have uniform expression of VGAT (**k**). However, gephyrin appears uniformly expressed (**l**). (**m**) We did not detect triple stained synapses. N = 3, n ≥ 9. (**n–s**) HCN1 staining suggests the region of the basket cell pinceau is unchanged from control (**n**) in basket cell mutant mice (**o**). Scale = 20 μm. N = 4, n ≥ 12. (**p–s**) The Purkinje cell axon initial segment (arrowheads) is obvious in control (**p,q**) and basket cell mutant mice (**r–s**) throughout the cerebellum with example images shown from both anterior and posterior lobules. Purkinje cell somas are indicated by asterisks. Scale = 10 μm. Per condition N = 3, n ≥ 9. (**a,b,h–s**) Dotted lines indicate the borders of the Purkinje cell layer (PCL) with the molecular layer (ML) above and the granular layer (GL) below.

We wondered whether removing GABAergic neurotransmission from molecular layer interneurons altered Purkinje cell function because of neurodegeneration in the cerebellum. This was important to test because dysgenesis during development and neurodegeneration in the adult are known to drive a number of electrophysiological abnormalities^3^. Specifically, alterations of the Purkinje cell dendrites after loss of interneuron connectivity would be a primary concern in our paradigm. We therefore measured molecular layer thickness as a proxy for dendrite span. Molecular layer thickness is a sensitive and straightforward measure for developmental and adult-associated defects that disrupt Purkinje cell dendrite size^32,45–47^. We stained sagittal cut tissue sections of the cerebellum with either anti-calbindin or anti-CAR8 antibody, which both mark Purkinje cells, and a fluorescent Nissl stain or DAPI, which outline all layers but heavily mark the granular layer because of the high cell density (Fig. 4a,b). Molecular layer thickness was assessed for lobule III/IV by measuring the perpendicular distance from the molecular layer-facing edge of a Purkinje cell soma to the outer edge of the molecular layer (basket cell control: N = 3, n = 9; basket cell mutant: N = 3, n = 9; stellate cell control: N = 3, n = 18; stellate cell mutant: N = 6, n = 36). We found that molecular layer thickness was not altered in either of the conditional mutant mice compared to the controls (Fig. 4c; stellate cells – control = 159.7 μm ± 9.20; mutant = 157.2 μm ± 5.5; *P* = 0.83; basket cells – control = 179.9 μm ± 3.83; mutant = 181.1 μm ± 3.7; *P* = 0.82). These data indicate that the outgrowth of the Purkinje cell dendritic tree during postnatal development, and its maintenance thereafter, were not adversely affected after we genetically silenced stellate cell and basket cell GABAergic output activity in the developing cerebellar cortex.

### Deleting *Vgat* in interneurons does not alter their targeting onto Purkinje cells

We next wanted to determine whether interneurons that lack *Vgat* are targeted to the correct regions of the Purkinje cell. We therefore examined whether the ultrastructure of synapses in the molecular layer was intact. To do so, we performed electron microscopy on sagittal sections cut through the adult cerebellum (stellate cell: N = 2, n ≥ 10, per condition; basket cell: N = 3, n ≥ 9, per condition). Using the distinctively large soma of Purkinje cells as a reference point for where the molecular layer starts, we assessed the integrity of inhibitory synapses in the Purkinje cell layer and molecular layer. Stellate cells terminate on the shaft of the Purkinje cell dendritic tree^34^. Excitatory synapses are distinguished from inhibitory synapses by the presence or absence, respectively, of a postsynaptic density that gives excitatory synapses an asymmetric appearance^34^. We observed synapses with symmetric morphologies that form postsynaptic terminals on the Purkinje cell dendrites in the molecular layer (Fig. 4d,e). These findings indicate that inhibitory synapses are retained in their correct positions within the cerebellar cortex despite the conditional silencing of GABAergic output at stellate cell interneuron synapses. We performed a similar analysis in mice with silenced basket cell output. The axons of several basket cells converge on single Purkinje cell somata to form the basket^34^. Basket cell axons extend further to form specialized pinceaux synapses around the axon initial segments of Purkinje cells^34,48,49^. We found inhibitory synapses on the Purkinje cell somata (Fig. 4f,g). Importantly, the interneuron synapses in the stellate cell and basket cell mutants contain distinct vesicles. This result indicates that despite the deletion of *Vgat* and the loss of GABAergic neurotransmission, the synaptic structural machinery that is required for housing neurotransmitters before release remains intact (Fig. 4d–g).

**Figure 5:**
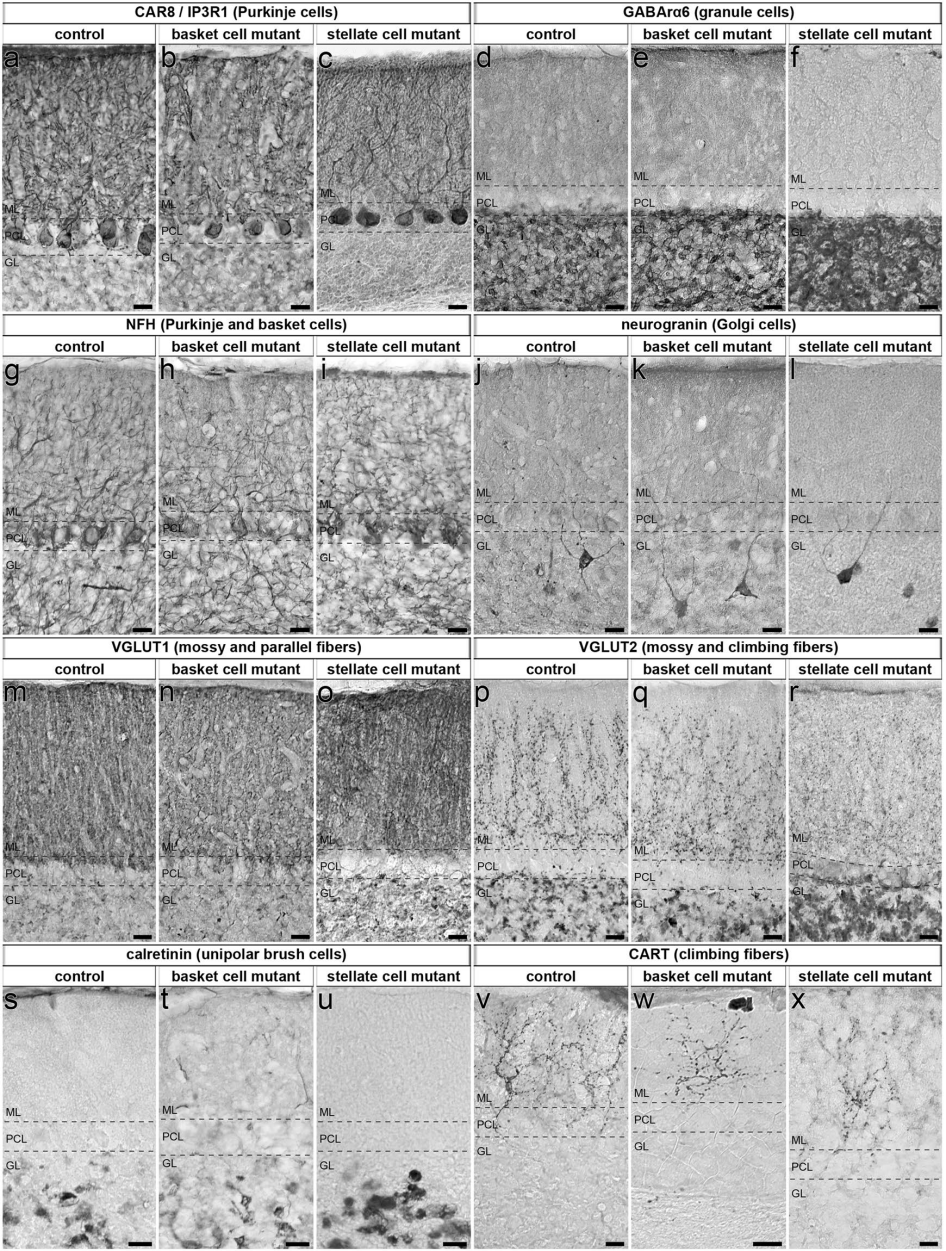
Conditional deletion of *Vgat* in molecular layer interneurons does not lead to gross cerebellar changes in cellular composition, cellular distribution, or layer patterning. (**a–x**) Cerebellar cell types were present and appeared unchanged in location and morphology despite the lack of VGAT in basket cells and stellate cells. Dotted lines indicate the borders of the Purkinje cell layer (PCL) with the molecular layer (ML) and the granular layer (GL). Scale = 20 μm. Control: N = 14, n ≥ 42; basket cell condition: N = 4, n ≥ 12; stellate cell condition: N = 6, n ≥ 18. (**a–c**) CAR8 and IP3R1. (**d–f**) GABAαR6. (**g–i**) NFH. (**j–l**) Neurogranin. (**m–o**) VGLUT1. (**p–r**) VGLUT2. (**s–u**) Calretinin. (**v–x**) CART.

To complement the electron microscopy studies in which we assessed presynaptic components, we also tested for the correct distribution of the postsynaptic structures belonging to the inhibitory synapses by immunohistochemical staining and light microscopy. Gephyrin is expressed in the postsynaptic compartment of inhibitory synapses^50^. In *Ascl1*^*CreER/*+^*;R26*^*fx-stop-EYFP*^*;Vgat*^*fx/fx*^ mutant mice treated with tamoxifen at P4, triple staining with gephyrin, VGAT, and GFP revealed a normal distribution of gephyrin in GFP-rich molecular layer regions that were devoid of VGAT expression (Fig. 4h–m). After silencing basket cells by giving tamoxifen at E18.5 to *Ascl1*^*CreER/*+^*;Vgat*^*fx/fx*^ mutants, we found that HCN1 (hyperpolarization-activated cyclic nucleotide-gated channel), which is expressed at both the pre-and post-synaptic sites at basket cell to Purkinje cell connections^51^, had a normal expression profile around the Purkinje cell layer (Fig. 4n,o). Moreover, we used AnkG (ankyrin-G) expression to show the presence of Purkinje cell axon initial segments after the loss of basket cell inhibitory neurotransmission in *Ascl1*^*CreER/*+^*;Vgat*^*fx/fx*^ mutant mice (Fig. 4p–s)^52^. We also sought to determine whether other major cell types of the cerebellar cortex were present as normal, since abnormal Purkinje cell activity may affect the gross organization of cerebellar circuitry. Purkinje cells, granule cells, Golgi cells, parallel fibers, mossy fibers, climbing fibers, and unipolar brush cells were all present with similar location and morphology in both the basket and stellate cell *Vgat* mutants as compared to control cerebella (Fig. 5; Control: N = 14, n ≥ 42; basket cell condition: N = 4, n ≥ 12; stellate cell condition: N = 6, n ≥ 18).

**Figure 6:**
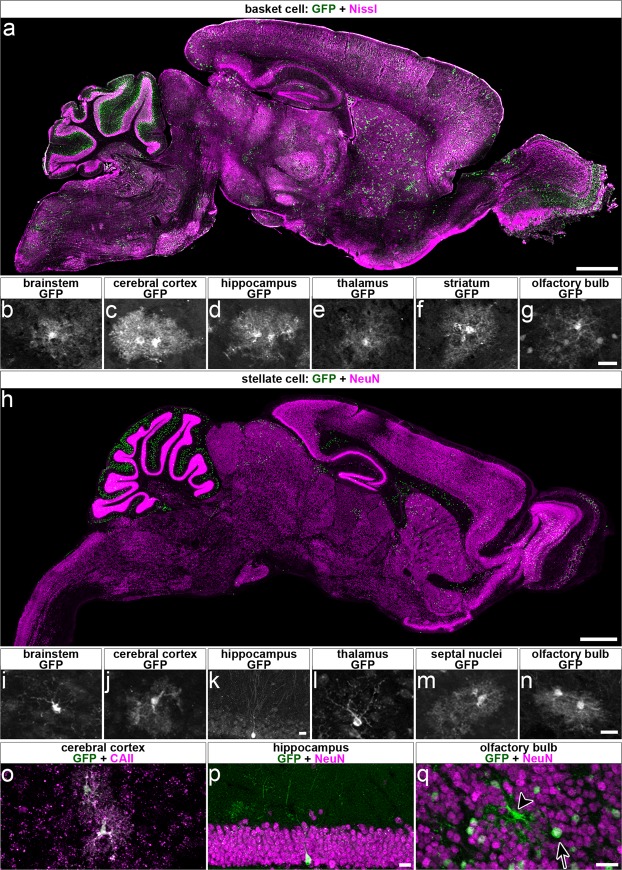
Conditional deletion of *Vgat* with *Ascl1*^*CreER*^ occurs in extracerebellar cell types that are unlikely to affect Purkinje cell activity in this manipulation. (**a**) Sparse labeling of cells occurs outside the cerebellum at the basket cell time point. Scale = 1 mm. (**b–g**) Many of the cells outside the cerebellum had morphologies that resembled glia, with the notable exception of cells in the olfactory bulb (**g**) where the majority of cells had the morphology of neurons, though cells with glial-like morphology were also present. Scale = 20 μm. (**h**) Sparse labeling outside of the cerebellum also occurred at the stellate cell time point. Scale = 1 mm. (**i–n**) While again many of the cells had morphologies that resembled glia, some cells with neuron-like morphologies were also detected. (**k**) Very sparse labeling of putative granule cells in the dentate gyrus occurred at the stellate cell time point, unlike at the basket cell time point at which no neurons were detected in the hippocampus. (**n**) Similar to the basket cell time point, many neurons in the olfactory bulb were labeled in addition to some glial-like cells. Scale = 20 μm. (**o**) Recombined cells with glial-like morphologies co-labeled with GFP and CAII, a maker of oligodendrocytes. Scale = 20 μm. N = 2, n ≥ 6. (**p**) Cells with neuron-like morphologies in the dentate gyrus of the hippocampus co-labeled with GFP and NeuN. Scale = 20 μm. N = 2, n ≥ 6. (**q**) Both neurons (arrow, co-labeled with GFP and NeuN) and glia (arrowhead, only labeled with GFP and not by NeuN) were labeled in the olfactory bulb. Scale = 20 μm. N = 2, n ≥ 6.

Finally, we sought to determine which *Ascl1* lineage cells had been manipulated outside of the cerebellum at both time points to determine whether it was likely the deletion of *Vgat* from these cells could result in the alterations in Purkinje cell firing that were found. Similar to previous work^22^, we found the majority of *Ascl1* lineage extracerebellar cells differentiating at both our basket and stellate time points give rise to oligodendrocytes and olfactory bulb neurons (Fig. 6). We therefore predicted that in our *Vgat* deletion paradigms, relatively few cells would have been manipulated outside of the cerebellum (Fig. 6a,h). Specifically, based on reporter expression we found that the majority of extracerebellar cells outside the olfactory bulb had a glial-like morphology (Fig. 6b–g,i,n). These putative glial cells were co-labeled with carbonic anhydrase II (CAII), suggesting their identity as oligodendrocytes (Fig. 6o; N = 2, n ≥ 6). The extracerebellar cells with a more neuron-like morphology included very sparse putative granule cells in the hippocampus that were detected only in the stellate cell marking scheme (Fig. 6k) and olfactory bulb neurons that were detected in both the stellate cell and basket cell marking schemes (Fig. 6g). The vast majority of the non-glial extracerebellar cells were found in the olfactory bulb. The identity of these cells as neurons was confirmed by the co-labeling of GFP reporter and NeuN (Fig. 6p,q; N = 2, n ≥ 6). These results indicate that the extracerebellar deletion of *Vgat* occurred in a population consisting largely of oligodendrocytes and olfactory bulb neurons, a population of neurons from which the loss of *Vgat* gene function would be highly unlikely to have significant effects on cerebellar Purkinje cell activity.

## Discussion

The cerebellum has served as the structure of choice in thousands of developmental, anatomical, functional, and behavioral studies. Among the reasons for its popularity are that its main cell types were identified more than a century ago^53^, and classic electrophysiological methods have allowed a detailed understanding of its connections^2,5,6,54^. However, it is still unclear how connectivity within the different classes of interneurons influences cerebellar cortical function. This study is focused on understanding whether the molecular layer interneurons have distinct inhibitory impacts on their target Purkinje cells. We tested how stellate cell and basket cell GABAergic neurotransmission influences Purkinje cell activity. To address this problem, we devised a genetic approach in which we used an *Ascl1*^*CreER*^ mouse line to delete the *Vgat* gene in the developing cerebellum. The *Ascl1*^*CreER*^ allele provided an opportunity for spatial and temporal manipulation of stellate cells independently from basket cells^21^. We found that the loss of *Vgat* in stellate cells altered the pattern of Purkinje cell simple spike firing and the rate of complex spike firing in alert mice, whereas deleting *Vgat* in basket cells changed the rate of both Purkinje cell simple spike and complex spike firing. The data suggest that molecular layer interneurons cooperate to establish Purkinje cell function *in vivo*.

Traditional high-resolution anatomy distinguishes molecular layer inhibitory interneurons based on multiple cellular, sub-cellular, and connectivity features^34^. Still, even using these various features it can be difficult to unambiguously assign neurons to a specific stellate cell or basket cell identity. Golgi staining analysis later suggested that classification based on distinct groups is challenging at best, since a more gradual and continuous identity could better reflect the molecular layer composition^29^. Analysis of gene expression yet again challenged the view, as the differential expression of multiple genes indicates at least some level of specificity and potentially unique identities within the interneurons^30^. Despite the differential expression, the authors also argue for a common origin. Indeed, stellate cells and basket cells arise from a common precursor pool in the ventricular zone^55^, and they are generated in waves from embryonic through postnatal development^18,21^. These different perspectives are further complicated by the observation that even though the somata are located in distinct positions within the dorsal-ventral axis of the molecular layer, there is some spread of both cell types’ somata into the middle molecular layer and a fuzzy separation of synaptic location (Fig. 2b,i,j). Regardless of anatomical or molecular distinctness, we asked whether any of these properties impact their contribution to cerebellar function. There is consensus that stellate cells and basket cells both synapse directly onto Purkinje cells^34^. But, do they influence Purkinje cells in a similar or different manner? To tackle this question, we used an *in vivo* genetic model in which fast GABAergic neurotransmission is blocked without causing neurodegeneration or overt circuit rearrangements that would, if present, alter Purkinje cell function. Genetic deletion of VGAT^23^, in general, does not impair the development of inhibitory synapses^56^. Nor does it alter the gross morphology or the basic structure of cerebellar circuits^32^. *In vivo* deletion of *Vgat* does, however, efficiently alter neuronal communication as evidenced by the depletion of inhibitory postsynaptic currents (IPSCs)^23,56–60^. Based on these slice and *in vitro* electrophysiology data acquired from the striatum, hippocampus, spinal cord, and retina, we predict similar effects for the cerebellum, and indeed our *in vivo* recordings support this notion. Our results uncover that stellate cells and basket cells do have distinct functional interactions with their Purkinje cell targets, with stellate silencing influencing Purkinje cell simple spike pattern and complex spike rate (Fig. 3g,i) and basket cell silencing altering the rate of both simple and complex spikes (Fig. 3m,q). However, our data cannot exclude the possibility that both cell types modulate multiple aspects of Purkinje cell function, even though each one might have a preferred interaction for modulating rate compared to pattern. In other words, there is likely no one molecular layer inhibitory cell type that is dedicated exclusively for the control of rate or the control of pattern. Our *in vivo* data are supported by work performed in slice, in which inhibitory activity was shown to control the regularity of interneuron firing^61^, and in a specific form of inhibitory rebound plasticity, basket cells were shown to control the pattern and rate of Purkinje cell output^31^. It would be interesting if basket cells and stellate cells are co-opted for rate versus pattern modulation depending on the specific behavioral task or the specific changes in plasticity that arise.

While the sheer size and complexity of the Purkinje cell and its dendritic arbor makes it difficult to predict how inhibitory postsynaptic potentials (IPSPs) propagate through the cell, one possibility is that the location of molecular layer interneuron subtypes’ synapses on the Purkinje cell could be in part responsible for their differential influence on Purkinje cell firing reported here. Dendritic attenuation as well as amplification mediated by voltage-gated ion channels in the Purkinje cell membrane could differentially affect IPSPs generated at synapses in the apical molecular layer compared to those formed around the Purkinje cell soma, theoretically creating spatial specificity for how Purkinje cell firing is controlled^62^. The frequency of IPSCs would likely be reduced in our manipulations, as they have been shown to be abolished in previous manipulations that have affected the entire molecular layer^61^. And, while a similar number of synapses were affected in each of our manipulations (Fig. 2o), because basket cells and stellate cells have different spontaneous firing rates^63^, it is possible that IPSCs were, by consequence, differentially affected by our manipulations. Similarly, the morphology of these cell types may play a role in determining their divergent effects on Purkinje cell activity. The unique morphology of the basket cell has been found to create a unique environment of extracellular voltage at the crucial region of the axon initial segment of Purkinje cells, allowing them to produce fast and robust inhibition of Purkinje cell activity^64^. This type of environment would not be present at stellate cell synapses. Based on our current data recorded *in vivo*, we can speculate that the predominant roles of the two classes of interneurons might be strengthened by network activity at the population level. Given the developmental nature of our manipulation, it is also possible the consequences we observed on Purkinje cell firing are due, at least in part, to compensatory or plasticity mechanisms after *Vgat* deletion. Although, compensatory mechanisms do not reliably recover functions after *Vgat* deletion^56,60^. Regardless, even if compensation was to have some impact in the cerebellum, it is still intriguing that Purkinje cell rate is refractory to the loss of GABAergic output from stellate cells whereas the pattern of activity is refractory to the elimination of GABAergic output from basket cells.

Stellate cells and basket cells do not function in isolation, and interactions within each cell type are not entirely random. Rather, the electrical and chemical connectivity in molecular layer interneuron populations are both highly structured, with connectivity clustering coefficients that reflect a spatial arrangement in the sagittal plane^65^. This architecture is intriguing because the entire cerebellum is organized around a map of sagittal compartments^66,67^. With specific importance to molecular layer interneuron circuitry, it is the Purkinje cells that determine all aspects of cerebellar sagittal organization. Purkinje cells are organized into a complex but precisely pleated array of sagittal compartments that are defined by cellular birth dates, lineage, gene expression, afferent connectivity, and neuronal firing properties^24,67^. Purkinje cell cues during development establish the fundamental map^68,69^ whereas Purkinje cell activity fine-tunes the topography into functional modules^32^. Molecular markers link subsets of interneurons to specific Purkinje cells forming zones defined by common expression^70^. There is some evidence that the inhibitory neurons specifically follow the expression of zebrinII^71^, the most extensively studied molecular marker of Purkinje cell zones^72,73^. Based on the *Ascl1*^*CreER*^ marking schemes for stellate cells and basket cells, there is no reason to believe that either paradigm marked cells that were restricted to particular zonal compartments (Fig. 2f,g), although it is possible that an interneuron’s birth date determines the particular zonal circuit that it will eventually wire into.

Deletion of *Vgat* using *Ascl1*^*CreER*^ was predicted to leave signaling intact in a substantial number of cells. By design, only subpopulations of molecular layer interneurons were targeted to achieve basket and stellate cell specificity. The majority of labeled cells were found in their canonical regions of the molecular layer with ~55% recombination in the basal molecular layer for the basket cell scheme and ~63% recombination in the apical molecular layer for the stellate cell scheme (Figs 1f,g, 2e). Similarly, VGAT was not entirely eliminated, but was instead reduced by ~57% in the basal molecular layer and ~64% in the apical molecular layer in the basket and stellate cell schemes, respectively (Fig. 2n,o). This efficiency is impressive for only a single dose of tamoxifen given that the molecular layer interneurons are born progressively over several embryonic and postnatal days. Still, even by creating a mosaic population of silenced interneurons we detected significant deficits in the overall function of Purkinje cells regardless of which particular cerebellar zone the recorded cell resided within^74,75^. Part of the reliability in producing Purkinje cells firing defects could be due to the connectivity of each manipulated interneuron, given that each one has the potential to make synaptic contacts with multiple Purkinje cells^34^. While stellate cells make mainly local synaptic connections with potentially fewer long-distance contacts, the basket cells could contact upwards of 9 Purkinje cells each^76^. The establishment of these distributions could also be altered in our genetic deletion paradigms. During development, synaptic activity controls the speed and direction of migration^77^. Because stellate cells and basket cells have intra- and inter-cellular connections with one another^34^, loss of GABAergic neurotransmission could impede neuronal migration. We suspect that if there were such deficits, they would likely be subtle or highly localized and specific since we did not detect obvious changes in cerebellar cell distribution by immunohistochemistry (Figs 2c, 4a,b, 5a–x) or afferent targeting as determined by electron microscopy (Fig. 4d–g).

Despite a long and rich history of understanding cerebellar cellular composition, circuitry, and function^2,4,53,78^, the last decade of cerebellar research has uncovered a number of additional cerebellar cortical afferent and efferent connections that could influence molecular layer interneuron processing. Purkinje cells not only contact the cerebellar nuclei, but through collaterals they also contact each other^79–83^, interneurons^83^, and granule cells^84^. The cerebellar nuclei indeed project out of the cerebellum, but they too also project back to the cerebellar cortex by way of inhibitory processes to Golgi cells and excitatory processes to Golgi cells^85^ and granule cells^86,87^. In this context, we should consider the various possible inputs to the molecular layer interneurons: climbing fibers to stellate cells and basket cells, Purkinje cells to stellate and basket cells, granule cells to stellate and basket cells, stellate cells to basket cells, basket cells to stellate cells, basket cells to basket cells, and stellate cells to stellate cells^34,83^. Before each interneuron communicates its output to its respective Purkinje cells, we also take into account that electrical connections tether rodent basket cells into groups of 5 and stellate cells in pairs^88^. Taking all these circuit features together, it is likely that this complex circuit structure lends itself to how basket cells and stellate cells integrate their inputs to have a differential effect on Purkinje cell firing. One has to ask, what are the functional consequences for such a circuit?

The differential modes of inhibition at the apical and basal portions of the molecular layer could serve to precisely gate the massive number of excitatory inputs from parallel fibers and the powerful input from climbing fibers onto Purkinje cells^89,90^. It is also possible that small-scale altered plasticity via indirect mechanisms could combine with the network-level features to instigate specific effects on climbing fiber spiking^91^. Moreover, the interaction between small patches of granule cells and Purkinje cells is shaped by molecular layer interneurons, and the strength of this inter-layer communication is dependent on relative position to the Purkinje cells in the sagittal and mediolateral axis^92^. It should also be considered that although the molecular layer interneurons are defined as GABAergic, they exhibit the expected inhibitory drive as well as a less appreciated excitatory influence^93^. Specifically, for our stellate cell silencing paradigm, it could be that the lack of a change in simple spike rate indicates an equilibrium rather than the absence of an effect. The predicted increase in Purkinje cell firing rate after loss of inhibitory GABA function would be countered by a decrease in Purkinje cell spikes after removing excitatory GABA function (Fig. 3). Under normal physiological conditions, such an effect could have a modulatory role in finely controlling Purkinje cell spike output, especially when dynamic changes are required during unrestricted behavior^94^. The impact of interneuron communication perhaps could also be appreciated at the population level. It could be that the local electrical networking together with their arrangement into rows facilitates a topographic interaction with zonally projecting climbing fibers from the inferior olive^95,96^. At the level of Purkinje cells, this ordered cellular and circuit architecture could manifest as synchronous activity^97^. Synchrony between chemically linked molecular layer interneurons has been reported^65^ and their impact is likely restricted to sagittal bands^98^. This is consistent with the long-standing hypothesis that synchronous neural activity promotes a level of neuronal ensemble dynamics that allow for muscles synergies to accommodate complex motor behaviors^99^. It is interesting to speculate that perhaps flexibility is built into the sagittal band networks because of unique basket cell to Purkinje cell and stellate cell to Purkinje cell communication, and their interaction with feedback from the inferior olive^100^. Loss of basket cell inhibition locally drives up Purkinje cell firing rate (Fig. 3), which theoretically could lead to a suppression of the cerebellar nuclei. Cerebellar nuclei neurons provide inhibitory output to the inferior olive^101^. It has been shown that removal of inhibition to the cerebellar nuclei can lead to a transient increase in nuclei neuron output called rebound firing^102,103^. In fact, with stronger inhibition of cerebellar nuclei firing, there is also stronger rebound potentials^102^. In our basket cell manipulation, wherein abnormally strong inhibition is likely present, it stands to reason that there may be abnormally strong rebound potentials at the level of the cerebellar nuclei. This would have the effect of stronger than usual inhibition of the inferior olive and a decrease in complex spike activity, similar to what we have observed (Fig. 3). For the loss of stellate cell inhibitory output, the increase in Purkinje cell local regularity (CV2; Fig. 3) could lead to an equivalent change in cerebellar nuclei pattern. Here, an increase in spike to spike regularity could reduce the impact of rebound activity^102,103^, which could have the effect of increasing olivary output – and an associated increase in complex spike activity (Fig. 3) – due to the lack of the transient increases in cerebellar nuclei output to the olive^104^. Ultimately, these interactions may be dependent upon the particular behavioral context and their impact perhaps even precisely modulated to accommodate the demands of the motor or non-motor cerebellar function.

## Conclusions

Cerebellar stellate cells and basket cells are the predominant cell type of the molecular layer. They arise from a common progenitor pool in the ventricular zone of the cerebellum and continue to divide and differentiate through postnatal development. We used an *Ascl1*^*CreER*^ genetic inducible allele to leverage this spatial and temporal pattern of development in order to manipulate the synaptic output of inhibitory interneurons. By blocking *Vgat* expression and then recording Purkinje cell activity in alert adult mice we uncovered that stellate cells establish the Purkinje cell simple spike firing pattern whereas basket cells determine their rate. Additionally, we found that Purkinje cell complex spike firing rate increases with a lack of stellate cell inhibition, but in contrast decreases with a lack of basket cell inhibition. This study establishes complementary roles for the GABAergic function of cerebellar molecular layer interneurons.

## Materials and Methods

### Mouse Lines

All experiments were performed according to a protocol approved by the Institutional Animal Care and Use Committee (IACUC) of Baylor College of Medicine. Three mouse lines were intercrossed to generate the various alleles. The first line expresses a knock-in construct of *CreER*^*T2*^ under the control of the *Ascl1* promoter (*Ascl1*^*CreER*^)^21^. The second line carries a knock-in floxed *Vgat* allele (*Vgat*^*fx*^)^23^. The third line expresses an enhanced yellow fluorescent protein (EYFP) knock-in construct with an upstream floxed transcriptional stop cassette, under the control of the *ROSA26* locus (*R26*^*fx-stop-EYFP*^)^105^. Our genotyping procedures for all of these alleles have been described before^32,38,47^. We bred the mice using standard timed pregnancies, and we designated noon on the day a vaginal plug was detected as embryonic day (E) 0.5 and the day of birth as P0. Mice of both sexes were studied. The mice were housed on a 14 h/10 h light/dark cycle.

### Cre induction

Tamoxifen (Sigma) was dissolved at 37 °C overnight in corn oil at a concentration of 20 mg/ml^33,38^. An 18-gauge syringe was used to pipette the solution up and down and dissolve any remaining tamoxifen particles. For targeting the stellate cells, tamoxifen was delivered at a dosage of 200ug/g into P4 postnatal pups by subcutaneous injection into the skinfold at the back of the neck. The pups were allowed to rest in a separate cage to prevent the mother from licking out the tamoxifen. After ~15 minutes, or once the subcutaneous bolus of tamoxifen solution had completely dispersed, each pup was returned to its home cage. For targeting the basket cells, 200ug/g tamoxifen was add-mixed with 50ug/g progesterone and administered to pregnant dams by oral gavage^106^.

### Immunohistochemistry

Perfusion and tissue fixation were performed as previously described^69^. Briefly, mice were anesthetized by intraperitoneal injection with Avertin (2,2,2-Tribromoethanol, Sigma-Aldrich Cat # T4). Cardiac perfusion was performed with 0.1 M phosphate-buffered saline (PBS; pH 7.4), then by 4% paraformaldehyde (4% PFA) diluted in PBS. For cryoembedding, brains were post-fixed in 4 °C for 24 to 48 hours in 4% PFA and then cryoprotected stepwise in sucrose solutions (15% and 30% diluted in PBS) and embedded in Tissue-Tek^®^ O.C.T. Compound (Sakura, Torrance, CA, USA). Samples were cut on a cryostat with a thickness of 40 μm and sections were collected as free-floating sections and stored in PBS. Immunohistochemistry procedures on free-floating frozen tissue sections were described previously^32,107–109^. After staining, the tissue sections were placed on electrostatically coated slides and allowed to dry.

### Cerebellar circuit markers

The integrity of the cerebellar circuitry was checked by determining the expression patterns of several synaptic and cell type-specific markers. Excitatory glutamatergic synapses contributed by granule cell parallel fibers were immunolabeled with rabbit anti-vesicular glutamate transporter 1 (anti-VGLUT1; 1:1000; Synaptic Systems, Göttingen, Germany). Excitatory synapses contributed by the mossy fibers in the granular layer^110^ and the climbing fibers in the molecular layer^111^ were immunolabeled with rabbit anti-VGLUT2 (1:500; Synaptic Systems, Göttingen, Germany; Cat. # 135 403) and rabbit polyclonal anti-cocaine- and amphetamine-related transcript peptide (CART; 1:250; Phoenix Pharmaceuticals, Burlingame, CA, USA; Cat. # H-003-62). The CART signal was amplified using a biotinylated secondary antibody (Vectastain Elite ABC method; Vector Labs; Burlingame, CA, USA) and used to visualize climbing fibers mainly in lobules IX and X^112^.

Purkinje cells were marked with anti-calbindin (1:1,000; Cat. # 300; Swant, Marly, Switzerland), rabbit polyclonal anti-carbonic anhydrase or CAR8 (CAVIII, 1:1000 l; Cat. # sc-67330, Santa Cruz Biotechnology), goat polyclonal anti-IP3R1 (1:500; Cat. # sc-6093, Santa Cruz Biotechnology, Dallas, TX, USA), goat polyclonal anti-RORα (1:250; Cat. # sc-6062, Santa Cruz Biotechnology, Dallas, TX, USA), and mouse monoclonal anti-ankyrin-G (1:200; Cat. # MABN466, clone N106/36, Millipore Sigma, Burlington, MA, USA). Purkinje cells and molecular layer interneurons were marked with rabbit polyclonal anti-parvalbumin (1:1000; Swant, Marly, Switzerland; Cat. # PV25). Excitatory interneurons were marked by rabbit polyclonal anti-calretinin (1:500; Swant, Marly, Switzerland; Cat. # CR7699/3 H). Granule cells were marked with rabbit polyclonal anti-gamma-aminobutyric acid receptor α6 (GABARα6; 1:500; Millipore Sigma, Burlington, MA, USA; Cat. # AB5610). Golgi cell interneurons in the adult cerebellum were marked by rabbit polyclonal anti-neurogranin (1:500; Millipore Sigma, Burlington, MA, USA; Cat. # AB5620)^113^. NeuN (1:250; Millipore Sigma, Burlington, MA, USA; Cat. #mab377) was used as a general neuronal marker and carbonic anhydrase II (CAII; BioRad, Hercules, CA, USA; Cat. # 00073) was used to label oligodendrocytes. Neuronal processes were also labeled with various markers. Mouse monoclonal anti-neurofilament heavy (NFH; also called anti-SMI-32; 1:1500; Covance, Princeton, NJ) immunolabeled the soma, dendrites, and axons of adult Purkinje cells, and the axons and terminals of basket cells. Mouse monoclonal anti-hyperpolarization-activated cyclic nucleotide-gated channel 1 (HCN1; 1:200; Alomone Labs; Jerusalem, Israel, Cat. # APC-056) was also used to label basket cell axons and pinceaux terminals. Guinea pig anti-gephyrin (1:500; Synaptic Systems, Göttingen, Germany, Cat. #147 004) was processed on paraffin embedded tissue cut at 10 µm. Some tissue sections were double-labeled with the different markers listed above plus chicken anti-GFP (1:1000; Abcam, Cambridge, UK, Cat. # AB13970) in order to visualize the EYFP reporter expression. The number of images that were analyzed for each condition is indicated with “n”, while the number of mice used is indicated with “N”. However, the overall analysis of lineage marked basket cells and stellate cells, which included a quantitative assessment of marking reliability and efficiency, structure of the marked cells, cell location, gross cerebellar anatomy, and co-expression with cell type specific markers, was conducted using 9 basket cell control mice, 6 basket cell mutant mice, 22 stellate cell control mice, and 25 stellate cell mutant mice.

For fluorescence immunostaining, we used Alexa-488, -555, and -647 secondary goat anti-mouse and anti-rabbit antibodies (1:1500, 1:1500, and 1:1000, respectively; Molecular Probes Inc., Eugene, OR, USA). For chromogenic immunostaining, we used horseradish peroxidase (HRP)-conjugated secondary goat anti-mouse or anti-rabbit antibodies (1:200; DAKO, Carpinteria, CA, USA). Antibody binding was revealed by incubating the tissue in the peroxidase substrate 3,3′-diaminobenzidine (DAB; Sigma-Aldrich, St Louis, MO, USA), which was made by dissolving a 100 mg DAB tablet in 40 ml PBS and 10 μL 30% H_2_O_2_. The DAB reaction was stopped with PBS when the optimal color intensity was reached. To preserve and contrast the fluorescence signal the tissue sections were mounted either with Fluoro-gel (Electron Microscopy Sciences, Hatfield, PA, USA) or a medium containing DAPI (Vectashield Antifade Mounting Medium with DAPI; Cat. # H-1200, Vector Laboratories, Burlingame, CA, USA).

### Imaging of immunostained tissue

Photomicrographs of the tissue sections were captured using Zeiss AxioCam MRm (fluorescence) and AxioCam MRc5 (DAB-reacted tissue sections) cameras mounted on a Zeiss Axio Imager.M2 microscope or on a Zeiss Axio Zoom.V16. Images of tissue sections were acquired and analyzed using either Zeiss AxioVision software (release 4.8) or Zeiss ZEN software (2012 edition). After imaging the tissue, the raw data were imported into Adobe Photoshop CS6 and corrected for brightness and contrast levels. The schematics were drawn in Adobe Illustrator CS6.

### VGAT quantification

We determined whether Cre induction deleted VGAT in interneurons by immunolabeling sagittal tissue sections from 1-month old mice with guinea pig anti-VGAT antibody (1:500; Synaptic Systems, Cat # 131 004; Göttingen, Germany). Images of the molecular layer were acquired with 20x magnification using Zeiss Axioimager microscope, in z-stack and ApoTome mode. Using the Fiji software for analysis, the background was subtracted using the built-in rolling ball method. The same settings were used for control and mutant tissue. The molecular layer was divided dorso-ventrally into three levels, and the levels were saved as regions of interest (ROI). The area and number of puncta in each level was measured using the built-in Analyze Particles function in Fiji and the density of VGAT-positive puncta for each level was calculated. Statistical significance at p < 0.05 was determined using the Student’s *t*-test. The number of images that were analyzed for each condition is indicated with “n”, while the number of mice used is indicated with “N”. Multiple lobules were analyzed from each image.

### Golgi-Cox staining

The brains were removed from the skull and then processed using the FD Rapid Golgi Stain Kit (PK 401 from FD Neurotechnologies, INC). All steps were carried out according to the manufacturer’s instructions. After staining, the tissue sections were dehydrated in a series of ethanol, cleared with xylene, and then mounted onto electrostatically coated glass slides with cytoseal. The tissue sections were allowed to dry before imaging.

### Molecular layer thickness measurement

Molecular layer thickness was measured from 3 mice per genotype in 3–4 sagittal sections spanning the midline per mouse, with a distance of ~80 μm in between each section. The tissues were immunostained with mouse monoclonal or rabbit polyclonal anti-calbindin (1:1,000; Cat. # 300; Swant, Marly, Switzerland) or anti-carbonic anhydrase to mark the Purkinje cell and molecular layers and NeuroTrace fluorescent Nissl stain (Life Technologies, Grand Island, NY, USA) or DAPI (Vectashield Antifade Mounting Medium with DAPI; Cat. # H-1200, Vector Laboratories, Burlingame, CA, USA) to mark the granular layer. The distance from the edge of the Purkinje cell soma to the apical edge of the molecular layer in the lobule III/IV region was measured using a line measurement tool from Fiji^114^. Measurements for each mouse were averaged and the numbers computed from each genotype were pooled and averaged again to obtain the mean molecular layer thickness. Statistical significance was defined as p < 0.05 using the Student’s *t*-test. The number of images that were analyzed for each condition is indicated with “n”, while the number of mice used is indicated with “N”.

### Electron Microscopy

Mice were anesthetized with Avertin and perfused with 0.9% room temperature saline, followed by an ice-cold solution of 4% paraformaldehyde and 2% glutaraldehyde in 0.1 M sodium cacodylate buffer (pH 7.4; 305–315 mOsm). Brains were harvested and cerebella were sagittally sectioned using a rodent brain matrix while immersed in fixative. The sections were transferred with fixative to a dish and the position of the molecular layer was noted. The region was chopped into pieces measuring less than 1 mm × 1 mm. The pieces were aspirated into a sample vial and fixed for 48 hours at 4 °C. Samples were treated with 1% Osmium tetroxide in 0.1 M cacodylate buffer for secondary fixation, then subsequently dehydrated in ethanol and propylene oxide and embedded in Embed-812 resin (Electron Microscopy Science, Hatfield, PA). Procedures were performed in a Ted Pella Bio Wave microwave oven with vacuum attachment. Tissues were cut with a Leica UC7 microtome into 50 nm ultra-thin sections and collected on Formvar-coated copper grids (Electron Microscopy Science, Hatfield, PA). Specimens were then stained with 1% uranyl acetate and 2.5% lead citrate and imaged using a JEOL JEM 1010 transmission electron microscope with an AMT XR-16 mid-mount 16 mega-pixel CCD camera. The images were imported into ImageJ where a smoothing function was applied and then the data were assembled in Adobe Photoshop CS6. The number of images that were analyzed for each condition is indicated with “n”, while the number of mice used is indicated with “N”.

### Surgery

Surgery for awake recordings was performed as detailed in White *et al*.^43^. Mice were sedated by gas anesthesia using 3% isoflurane, then injected with a ketamine-dexmedetomidine cocktail at a dosage of 80/16 mg/kg, respectively. They were then transferred from the anesthesia chamber to a stereotaxic platform (David Kopf Instruments, Tujunga, CA, USA) and head-fixed with metal ear bars. Sterile surgery techniques were followed. A custom-made headplate was first attached to the Bregma region using Metabond. This headplate was used to affix the mouse’s head to the awake recording apparatus. After the adhesive has dried, a small hole slightly smaller than a 1/16 screw (00-90 × 1/16 flat point stainless steel machine screws #B002SG89QQ) was drilled to the left of the cerebellar midline. Drilling was stopped before the skull was completely penetrated. An ethanol-sterilized 1/16 screw, which served as an anchor for dental cement, was secured into the drillhole with a screwdriver until it was tightly in place. Another craniotomy was performed on the right side of the midline. A hole approximately ~5 mm in diameter was drilled, taking care not to damage the dura. Once the craniotomy was complete, the hole was covered in triple antibiotic ointment to prepare for the installation of the recording chamber. A piece of straw with a 5–7 mm diameter and a height of 4–5 mm was ethanol-sterilized and air-dried. One end of the straw was dipped in Metabond and carefully placed on top of the craniotomy. Once the adhesive was dry, dental cement (A-M Systems dental cement powder #525000 and solvent #526000) was applied on the outer edge of the straw to fill in holes and to further secure the chamber. After the dental cement had dried, a fresh layer was applied around the straw and the Bregma region where the headplate was attached. After the layer dried, a final layer was applied throughout the site of surgery, including the screw, the top and underside of the headplate, and along the edges of the straw. The skin surrounding the site of the surgery was fixed to the dental cement using 3M Vetbond (#NC0304169) after the cement has completely dried. While the last layer of dental cement was drying, 0.6 mg/kg buprenorphine was injected subcutaneously as an analgesic. After the surgery, the mouse was placed in a warming box (V500, Peco Services Ltd., Cumbria, UK) to prevent hypothermia while the anesthesia wears off. Once the mouse was awake and mobile, it was returned to the home cage. The mouse was allowed to recover for 2–3 days and was given buprenorphine every 6–12 hours. On the third day, training on the running wheel was started. Training sessions were done twice a day for 30 minutes. Before recording, the antibiotic ointment in the chamber was removed using a compressed foam-tipped swab (Cleanfoam^®^ Swab) and replaced with 0.9% w/v NaCl solution. After each recording session, the solution was removed with a cotton tip or by aspiration with a micropipette and fresh antibiotic ointment applied.

### *In vivo* electrophysiology

Single-unit extracellular recordings were performed as described previously^46,47,115^. During the recordings, the reference electrode tip was immersed in the saline chamber. Tungsten electrodes (Thomas Recording, Giessen, Germany) with an impedance of 5–8 MΩ were controlled from a headstage using a motorized micromanipulator (MP-225; Sutter Instrument Co., Novato, CA, USA). Signals were acquired using an ELC-03XS amplifier (NPI Electronic Instruments, Tamm, Germany) with band-pass filter settings of 0.3–13 kHz. Analog signals were digitized using a CED Power 1401 and stored and analyzed using Spike 2 software (CED, Cambridge, UK).

Purkinje cells were recorded at a depth of approximately 0–2 mm from the tissue surface while the mouse was alert and standing during quiet wakefulness on a foam wheel. Purkinje cells were identified by the unique presence of two types of action potentials: simple spikes, which are intrinsically generated, and complex spikes, which are generated by climbing fiber input. Neurons from which we obtained clear, continuous recordings lasting 200–300 seconds were included in the analysis. Analysis of firing properties was performed using Spike2, MS Excel, and GraphPad Prism. Firing rate (Hz) was calculated as the number of spikes recorded over a given time period. Coefficient of variance, or CV, was calculated as the ratio of the standard deviation of the interspike intervals (ISI) over the mean ISI. CV2 was calculated with the formula (CV2 = 2|ISI_n+1_ − ISI_n_|/(ISI_n+1_ + ISI_n_)), as described previously^44^. Purkinje cell simple spike and complex spike activity was sorted, analyzed, and data reported as mean ± standard error of the mean (SEM). GraphPad Prism ROUT method of outlier detection was used with Q = 1% to remove outlier cells before further analysis. Statistical analyses were performed with unpaired, two-tailed Student’s t-tests. Statistical significance is indicated in the graphs as *P < 0.05, **P < 0.01, ***P < 0.001, ****P < ***0.0001. The number of Purkinje cells that were analyzed for each measurement is indicated with “n”, while the number of mice recorded for each genotype analyzed is indicated with “N”.

## Data Availability

All data generated or analyzed during this study are included in this published article.
